# Endocytosis and serpentine filopodia drive blebbishield-mediated resurrection of apoptotic cancer stem cells

**DOI:** 10.1038/cddiscovery.2015.69

**Published:** 2016-01-25

**Authors:** G G Jinesh, A M Kamat

**Affiliations:** 1 Department of Urology, The University of Texas MD Anderson Cancer Center, Houston, TX, USA

## Abstract

The blebbishield emergency program helps to resurrect apoptotic cancer stem cells (CSCs) themselves. Understanding the mechanisms behind this program is essential to block resurrection of CSCs during cancer therapy. Here we demonstrate that endocytosis drives serpentine filopodia to construct blebbishields from apoptotic bodies and that a VEGF-VEGFR2-endocytosis-p70S6K axis governs subsequent transformation. Disengagement of RalGDS from E-cadherin initiates endocytosis of RalGDS and its novel interaction partners cdc42, VEGFR2, cleaved *β*-catenin, and PKC-*ζ* as well as its known interaction partner K-Ras. We also report novel interactions of p45S6K (cleaved p70S6K) and PKM-*ζ* with PAK-1 filopodia-forming machinery specifically in blebbishields. Thus, a RalGDS-endocytosis-filopodia-VEGFR2-K-Ras-p70S6K axis drives the blebbishield emergency program, and therapeutic targeting of this axis might prevent resurrection of CSCs during cancer therapy.

## Introduction

Blebbishield formation is fundamental to resurrect cancer stem cells (CSCs) after apoptosis. Apoptotic CSCs reconstruct apoptotic bodies into blebbishields, which then fuse with one another to form stem-cell spheres (transformation);^1^ however, the cellular and molecular events underlying the reconstruction of apoptotic bodies into blebbishields and subsequent transformation from blebbishields are not understood. CSCs have been implicated in resistance to therapy.^2^ Understanding the mechanisms of blebbishield emergency program is essential to block resurrection of CSCs during cancer therapy to prevent recurrence.^3^

Mitotic cells resemble apoptotic cells with respect to Golgi fragmentation^4,5^ despite the survival outcomes of mitotic cells and apoptotic cells are totally different. Mitotic cells transiently arrest endocytosis during mitosis and resume endocytosis during the reattachment phase,^6–9^ whereas in apoptotic cells, endocytosis is inactivated.^10^ Since blebbishields are capable of survival after apoptosis, blebbishields might use mechanisms similar to mitosis to enable survival. Recent evidence demonstrates that RalA and RalA binding protein 1 (RalBP1; also known as RLIP76) are necessary for Ras-induced tumorigenesis in human cells,^11^ where RalBP1 mediates endocytosis.^12^ RalBP1 is regulated by RalA, which in turn is super-regulated by RalGDS.^13^ Furthermore, endocytosis is an inevitable feature of tumorigenesis.^14^ Hence, endocytosis is a platform that could connect mitotic cells, blebbishields, and tumorigenesis.

Here we demonstrate that blebbishields and mitotic cells expose their endomembranes, such as Golgi membranes, at the cell surface and resorb these exposed membranes back to the perinuclear region by endocytosis (endocytosis in blebbishields is in contrast to apoptosis). Endocytosis drives novel serpentine filopodia formation, which tether and tie apoptotic bodies together to facilitate blebbishield formation. Inhibiting dynamin-mediated endosome scission blocks blebbishield formation and promotes apoptotic body formation. On the other hand, the VEGF-VEGFR2-endocytosis-p70S6K axis is required for blebbishield-mediated transformation. Apoptotic events release RalGDS and its novel interaction partners cdc42, VEGFR2, cleaved *β*-catenin, and PKC-*ζ*, as well as its previously known interaction partner K-Ras, from E-cadherin-mediated plasma membrane anchorage to initiate endocytosis in blebbishields. Furthermore, we detected novel p45S6K (cleaved p70S6K) and PKM-*ζ* (cleaved PKC-*ζ*) interactions with PAK-1 filopodia-forming machinery specifically in blebbishields, explaining formation of serpentine filopodia. Therefore, the RalGDS-endocytosis-filopodia-VEGFR2-K-Ras-p70S6K axis is essential for blebbishield-mediated resurrection of CSCs, and therapeutic targeting of this axis might prevent resurrection of CSCs during cancer therapy.

## Results and discussion

### Fusogenic membranes from the perinuclear region mediate fusion of blebbishields with mitotic cells

**Figure 1:**
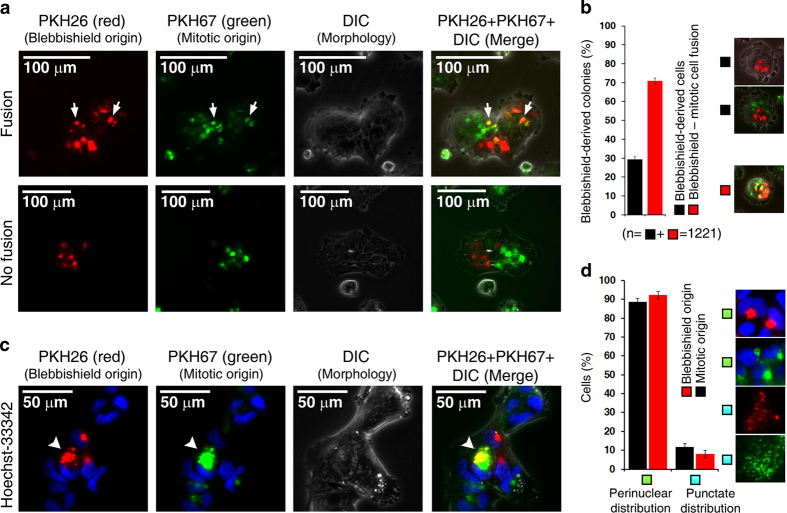
Fusogenic membranes from the perinuclear region mediate fusion of blebbishields with mitotic cells. (**a**) Surface membranes of blebbishields and mitotic cells labeled with PKH dyes accumulated at the perinuclear region after reattachment to the substratum. Arrows indicate fusion of blebbishields with mitotic cells. (**b**) Quantification of blebbishield–mitotic cell fusion. n, number of colonies. (**c**) Blebbishield surface membranes were restricted to the *trans* perinuclear region, whereas mitotic-cell surface membranes occupied both the *cis* and *trans* perinuclear regions (arrowheads). DIC, differential interference contrast. (**d**) Blebbishield and mitotic-cell surface membranes exhibited fragmentation and perinuclear reassembly phenotypes similar to those of Golgi membranes.

Mitotic cells resemble apoptotic cells in two major aspects: loss of attachment with adjacent cells and Golgi fragmentation.^4,5^ However, mitotic cells exhibit more efficient reattachment to the substratum than apoptotic cells do; hence, blebbishields might use a similar mechanism to resurrect after apoptosis. Since blebbishields exhibit intense fusion behavior,^1^ we first examined whether blebbishields can fuse with mitotic cells by simultaneously tracking the origin of surface membranes of blebbishields and mitotic cells. We tagged the surface membranes of blebbishields with PKH26 (red) and the surface membranes of mitotic cells with PKH67 (green) and allowed the blebbishields and mitotic cells to reattach to the substratum in co-culture. Interestingly, 70.79% (S.E.M.±1.58%) of blebbishield-derived cells fused with mitotic cells (Figures 1a and b). Co-existence of blebbishield-derived cells and mitosis-derived cells in the same colonies without fusion demonstrated that the PKH dyes and dye-tagged membranes were not cross-presented to adjacent cells by any means (Figure 1a). Importantly, both blebbishield surface membranes and mitotic-cell surface membranes accumulated at the perinuclear region in reattached cells, albeit with minor differences in the *cis* to *trans* perinuclear distribution (similar to *cis*-Golgi to *trans*-Golgi positions; Figure 1c). Time-lapse microscopy revealed vesicular movements around perinuclear accumulations, suggesting that the PKH-labeled membranes were transported as vesicles (Supplementary Movie S1). Notably, 92.1% (S.E.M.±1.96%) of blebbishield-derived cells and 88.4% (S.E.M.±2.12%) of reattached mitotic cells exhibited perinuclear accumulation of PKH-labeled membranes (which had previously been exposed at the cell surface in blebbishields and in mitotic cells, respectively); the remaining cells exhibited punctate distribution of PKH-labeled surface membranes throughout the cytoplasm, similar to the distribution of fragmented Golgi vesicles (Figure 1d).

### Serpentine filopodia and box-braid filopodia network provide mechanical force to construct blebbishields from apoptotic bodies

**Figure 2:**
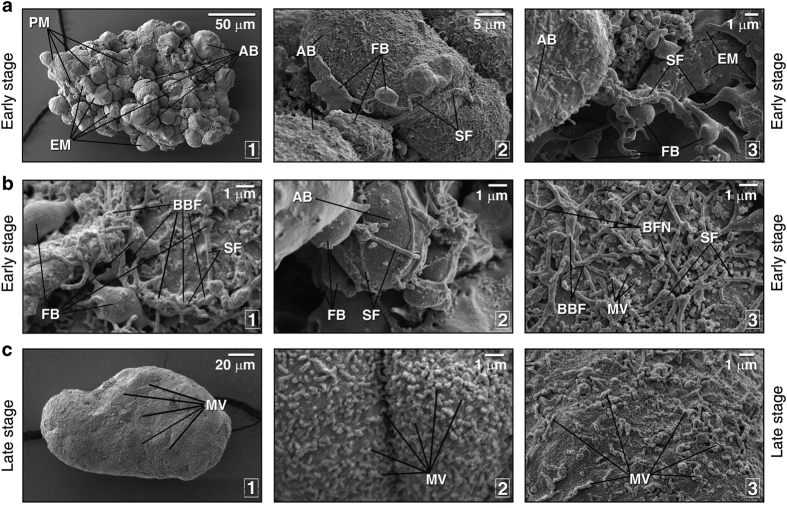
Early-stage blebbishields are formed by serpentine and box-braid filopodia, whereas in late-stage blebbishields, filopodia are reduced to microvilli. (**a1**) Scanning electron microscopy of early-stage blebbishields showed two distinct types of apoptotic bodies (AB), one type with endomembranes (EM) and the other with a plasma membranes (PM). (**a2–3**) Serpentine filopodia (SF) with a filopodial bulb (FB) provided initial contact between apoptotic bodies (AB). (**b1–3**) Serpentine filopodia (SF) with a filopodial bulb (FB) formed box-braid filopodia (BBF), formed a BBF network (BFN), and tied apoptotic bodies (AB) together to provide mechanical support for blebbishield construction. MV, microvilli. (**c1-3**) Late-stage blebbishields had fewer or no serpentine or box-braid filopodia but had smaller microvilli (MV).

Unlike classic apoptotic cells, in which cellular contents are packed under the plasma membrane into apoptotic bodies, early-stage blebbishields displayed two distinct types of apoptotic bodies, one with a rough plasma membrane surface and the other with a relatively smooth endomembrane surface (Figure 2a, subpanel 1). Interestingly, in early-stage blebbishields, both of these types of apoptotic bodies were tethered by long (>5 *μ*m), stout (∼0.25 *μ*m in diameter), serpentine filopodia (Figure 2a, subpanels 2 and 3, and Figure 2b). Serpentine filopodia of blebbishields differed from previously described long filopodia^15,16^ in that serpentine filopodia possessed a bulb (Figure 2a, subpanels 2 and 3; and Figure 2b, subpanels 1 and 2), which might supply the cytoskeletal and energy components needed to extend and operate the serpentine filopodia. The serpentine filopodia tethering adjacent apoptotic bodies in early-stage blebbishields were twisted on each other to form box-braid filopodia (Figure 2b, subpanels 1 and 3) and formed box-braid filopodia network to cover the surface of the apoptotic bodies (Figure 2b, subpanel 3). The finding of serpentine filopodia tied around apoptotic bodies (Figure 2b, subpanel 2) indicated that serpentine filopodia provided mechanical strength to hold the apoptotic bodies together by apposition. Filopodia formation is one of the early steps of membrane fusion between cells^17^ where filopodia probe the environment to find target membranes and form adherent junctions by ‘adhesion-zippers’ to facilitate fusion.^16,18^ Adhesion-zippers are anti-parallel filopodia pairs from adjacent cells with *β*-catenin-*α*-catenin linkers between surface E-cadherin and the internal actin cytoskeleton.^18^ Serpentine filopodia differed from adhesion-zippers in that serpentine filopodia formed box-braid filopodia (not anti-parallel), formed box-braid filopodia networks, and also possessed bulbs. In contrast to early-stage blebbishields, late-stage blebbishields had relatively smoother surfaces with less or no endomembrane exposure and had microvilli (shorter filopodia, <1 *μ*m in length; Figure 2c). These data strongly supported a mechanical role of filopodia in constructing blebbishields from apoptotic bodies.

### Endocytosis mediates surface-to-perinuclear trafficking of exposed endomembranes in blebbishields and mitotic cells

**Figure 3:**
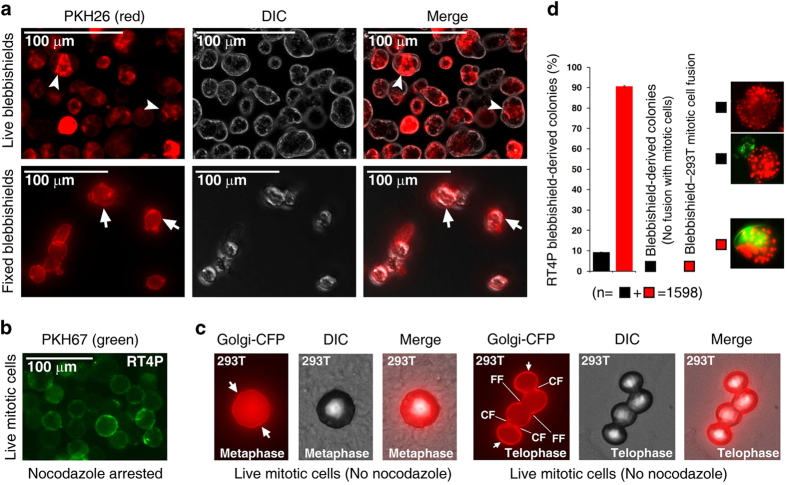
Blebbishields rapidly internalize fusogenic membranes and mitotic cells fuse to each other using surface-exposed Golgi membranes. (**a**) Live blebbishields internalized labeled surface membranes (arrowheads), but fixed blebbishields did not. Note the labeling is restricted to the surface (arrows). DIC, differential interference contrast. (**b**) Nocodazole-arrested mitotic cells did not internalize surface membranes. (**c**) Genetic labeling of Golgi membranes using Golgi-CFP revealed that they were exposed to the cell surface (arrows) during metaphase and telophase in untreated 293T mitotic cells and underwent fusion during telophase. CF, mitotic cleavage furrow; FF, fusion furrow. DIC, differential interference contrast. (**d**) 293T mitotic cells were capable of fusion with blebbishields derived from RT4P cells. n, number of colonies.

Interestingly, endocytosis is known to drive filopodia formation.^19^ Hence, endocytosis might help to resorb endomembranes as well as promote filopodia formation as a positive feed-forward loop. To test this concept, we first examined the extent of endocytosis in blebbishields and mitotic cells using PKH26 and PKH67, respectively, as membrane tracers. In live blebbishields, a 5-min pulse with PKH26 led to rapid accumulation of PKH26-labeled membranes in the blebbishield interior, whereas in fixed blebbishields, after a 5-min pulse with PKH26, the PKH26-labeled membranes were largely restricted to the blebbishield surface, indicating the existence of robust endocytosis in live blebbishields (Figure 3a). On the other hand, live mitotic cells (nocodazole arrested) had uniform surface labeling of PKH67, indicating less or no endocytosis in mitotic cells (Figure 3b). This finding is in agreement with the fact that mitotic cells transiently arrest endocytosis during mitosis and resume endocytosis during the reattachment phase.^20^ To rule out the possibility that use of nocodazole to arrest mitotic cells impeded endocytosis by targeting the cytoskeletal network, we genetically labeled the Golgi membranes of 293T cells and studied the surface localization and fusion behavior of these Golgi membranes in mitotic cells. During metaphase, Golgi membranes were distributed at both the cell surface and cell interior, whereas during telophase, Golgi membranes were predominantly localized at the cell surface and at the cleavage furrow (Figure 3c). Interestingly, 293T mitotic cells at telophase exhibited fusion with adjacent mitotic cells at telophase by forming fusion furrow, demonstrating that the fusogenic behavior of mitotic cells is derived from Golgi membranes (Figure 3c). This finding agrees with the previously reported finding that mitotic cells at telophase and anaphase attached to each other, with filopodia and desmosomes shown by transmission electron microscopy.^21^ We further found that 90.68% (S.E.M.±0.35%) of blebbishields derived from RT4P cells (human bladder cancer cell origin) fused with 293T mitotic cells (human kidney cell origin), demonstrating that blebbishields were able to fuse with mitotic cells from a different tissue of origin (Figure 3d).

### Inhibition of endocytosis or Golgi reassembly abrogates blebbishield formation and transformation from blebbishields

**Figure 4:**
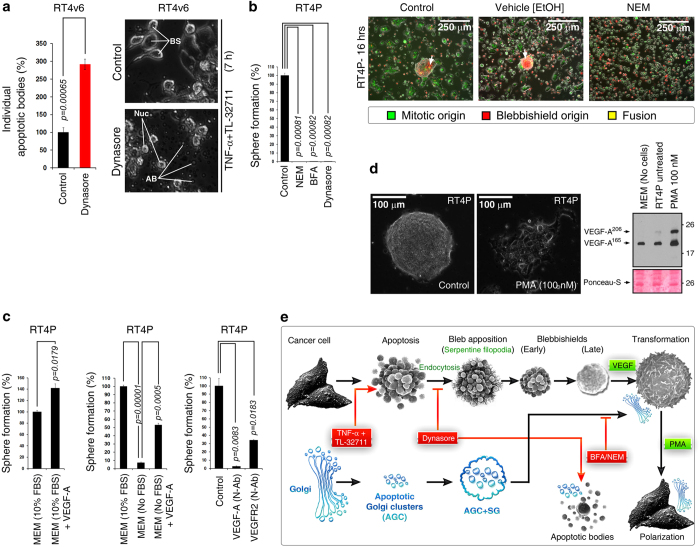
Endocytosis, VEGF signaling, and Golgi integrity drive blebbishield formation and transformation from blebbishields. (**a**) Blocking endocytosis by dynasore promoted formation of individual apoptotic bodies and prevented formation of blebbishields (also see Supplementary Movie S2). BS, blebbishields; AB, apoptotic bodies. (**b**) Inhibitors of endocytosis and Golgi integrity blocked transformation from blebbishields (left panel). NEM, *N*-ethylmaleimide; BFA, brefeldin-A. NEM blocked reattachment of both blebbishields (PKH26 (red)) and mitotic cells (PKH67 (green)) to substratum (right panel). (**c**) Isolated blebbishields formed more spheres with exogenous VEGF (left panel). Serum withdrawal reduced transformation from blebbishields, and exogenous VEGF rescued transformation (middle panel). Addition of 1 *μ*g/ml neutralizing antibodies (N-Ab) to VEGF or VEGFR2 blocked transformation from blebbishields in 10% MEM (right panel). (**d**) Phorbol-myristic acetate (PMA) accelerated polarization of cells from blebbishield-transformed spheres (also see Supplementary Movie S4; photomicrograph). PMA enhanced VEGF secretion detected by Western blotting of conditioned media (right panel). Note that VEGF is present in FBS of MEM (lane-1). (**e**) Schematic showing how endocytosis drives blebbishield formation. The status of Golgi fragmentation is depicted in blue. AGC, apoptotic-Golgi clusters; SG, surface Golgi membranes. Note: Though Golgi membranes are at the surface of blebbishields (based on PKH26 staining), transmission electron microscopy indicates that internally, Golgi remains as AGC.^1^

Endocytosis is known to drive filopodia formation.^19^ To confirm that endocytosis is required for blebbishield formation, we used dynasore, which inhibits the scission of endocytic vesicles from the plasma membrane by inhibiting dynamin to block endocytosis.^22,23^ We generated blebbishields by treating RT4v6 cells with TNF-*α* in combination with Smac mimetic TL-32711 for 7 h in the presence or absence of dynasore (we previously reported that the combination of TNF-*α* and Smac mimetic induces apoptosis in RT4v6 cells^24,25^). Inhibition of endocytosis increased apoptotic body formation by 191% (S.E.M.±13.4%) than the number of apoptotic bodies formed in control cells (Figure 4a). Furthermore, inhibition of endocytosis inhibited blebbishield formation in RT4P cells (Supplementary Movie S2).

We next examined whether Golgi integrity/reassembly inhibitors or dynasore could influence transformation from blebbishields. For this purpose, we used Golgi integrity inhibitors and endosomal traffic inhibitors *N*-ethylmaleimide^26^ and brefeldin-A^27^ and endocytosis inhibitor dynasore to test sphere formation (transformation) from blebbishields. Interestingly, *N*-ethylmaleimide, brefeldin-A, and dynasore were all able to completely abolish sphere formation from blebbishields (Figure 4b). Notably, NEM was able to inhibit reattachment of both blebbishields and mitotic cells, demonstrating that blebbishields and mitotic cells use a similar mechanism for reattachment (Figure 4b). Taken together, these data demonstrated that endocytosis-driven filopodia formation is necessary to construct blebbishields from apoptotic bodies and to override apoptotic body formation (Figure 4e) and that Golgi reassembly is required for transformation.

### VEGF signaling drives transformation from blebbishields

We previously implicated VEGF signaling in transformation from blebbishields.^1^ Here we examined whether VEGF signaling is required for transformation from blebbishields. Exogenous VEGF-A enhanced transformation from RT4P derived blebbishields by 42% (S.E.M.±7.3%) in the presence of FBS (Figure 4c; FBS contains VEGF-A; see Figure 4d). FBS withdrawal reduced transformation from blebbishields by 92.7% (S.E.M.±0.84%), whereas exogenous VEGF-A in the absence of FBS rescued transformation by 46% more (SEM±2.1%) compared with 7.3% in the FBS withdrawn condition (Figure 4c). Furthermore, neutralizing antibodies against VEGF-A and VEGFR2 reduced transformation from blebbishields by 97.5% (S.E.M.±0.7%) and 65.8% (S.E.M.±0.5%), respectively (Figure 4c), demonstrating that VEGF signaling drives transformation from blebbishields. However, VEGF neutralizing antibody could not inhibit or slow down reattachment of trypsinized RT4P cells, demonstrating that the role of VEGF signaling in attachment is specific to blebbishields (Supplementary Movie S3). Furthermore, phorbol-myristic acetate (PMA) enhanced recovery from the transformed sphere phase to polarized cancer cells and augmented VEGF-A autocrine production (Figure 4d and Supplementary Movie S4). Taken together, these data demonstrated that transformation from blebbishields is driven by VEGF signaling (Figure 4e).

### VEGFR2 and syntaxin-6 colocalize with internalized PKH26-labeled blebbishield surface membranes

**Figure 5:**
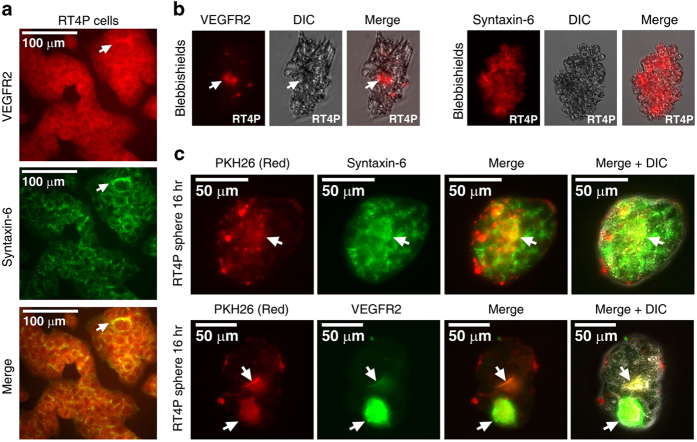
VEGFR2 undergoes endocytosis in blebbishields, and internalized VEGFR2 colocalizes with PKH26-labeled lipid membranes in transformed spheres. (**a** and **b**) Double immunofluorescence analysis of VEGFR2 and syntaxin-6 (*trans*-Golgi-network marker) showed their co-localization at the perinuclear *trans*-Golgi-network (arrows) in non-apoptotic cells (**a**) Immunofluorescence analysis of VEGFR2 and syntaxin-6 and in freshly fixed blebbishields; internalized VEGFR2 is marked by arrows (**b**). (**c**) Lipo-immunofluorescence analysis of VEGFR2 and syntaxin-6 co-localization with PKH26-labeled blebbishield surface membrane lipids (internalized in spheres; co-localization marked by arrows). DIC, differential interference contrast.

VEGFR2 is a major signal transduction receptor for VEGF-A. VEGFR2 shuttles between the plasma membrane and the *trans*-Golgi-network with the help of syntaxin-6.^28^ Hence, we examined the localization of VEGFR2 and syntaxin-6 in intact RT4P cells. We found that they colocalized at the perinuclear region (Figure 5a). Immunofluorescence analysis revealed that VEGFR2 was internalized in late-stage blebbishields and syntaxin-6 was localized throughout the blebs in early-stage blebbishields (Figure 5b). We further confirmed that VEGFR2 and syntazin-6 colocalized with PKH-26 labeled membranes in transformed spheres by lipo-immunofluorescence analysis (Figure 5c). These results confirmed that VEGFR2 is trafficked from the blebbishield surface to the trans-Golgi network at the perinuclear region by endocytosis (PKH data from Figure 1 compared with Figure 5c).

### Endocytosis of VEGFR2 and *β*-catenin is triggered by disengagement of RalGDS from E-cadherin

Although the data so far demonstrated the role of VEGF signaling and endocytosis in blebbishield formation and transformation from blebbishields, the molecular drivers were elusive. To track the molecular drivers, we looked for common regulators of endocytosis, filopodia formation, VEGFR2 signaling, PMA signaling, tumorigenesis, cell fusion, and transformation. RalGDS-RalA-RalBP1 signaling regulated most of these processes, including Ras-induced tumorigenesis,^11,29^ endocytosis,^12^ filopodia formation,^30,31^ and membrane fusion by fusogenic lipid synthesis,^32^ although it did not regulate VEGFR2 signaling or PMA signaling. Hence, we examined the links between these multiple processes using RalGDS interaction assay in blebbishields, mitotic cells, and non-blebbishield cells (blebbishield depleted cells).

**Figure 6:**
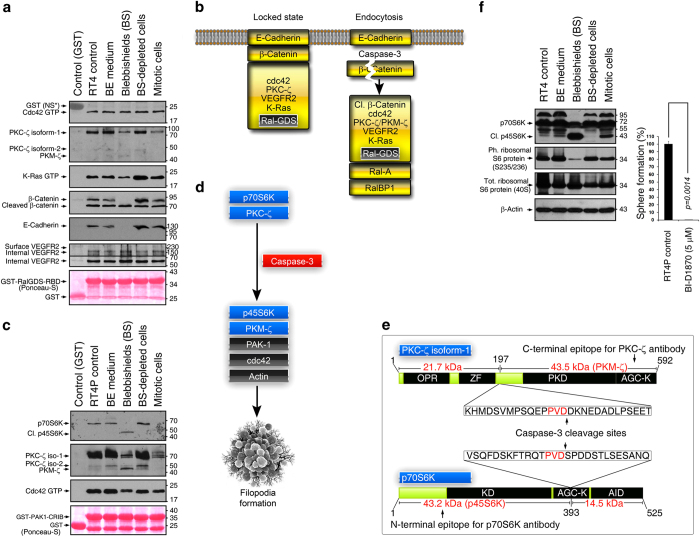
Novel protein interactions reveal regulators of endocytosis and filopodia formation in blebbishields. (**a**) GST pulldown using RalGDS-RBD as bait showed constitutive interactions of cdc42, PKC-*ζ*, K-Ras, full-length and cleaved *β*-catenin, and internal VEGFR2 with E-cadherin in RT4P cells; interactions with E-cadherin and full-length *β*-catenin were lost in blebbishields. Note: the blebbishield lane and blebbishield (BS)-depleted cells lane are fractions of the blebbishield ejection (BE) medium lane. (**b**) Proposed model of E-cadherin-mediated locked state and how caspase-3 cleavage of *β*-catenin could release RalGDS and interaction partners from E-cadherin-mediated lock. Cl., cleaved. (**c**) Selective interaction of p45S6K (caspase-3-cleaved p70S6K) and PKM-*ζ* (cleaved PKC-*ζ*) with PAK-1-CRIB domain along with filopodia initiator cdc42. Cl., cleaved; iso, isoform. (**d**) Caspase-3-mediated cleavage of p70S6K and PKC-*ζ* to generate p45S6K and PKM-*ζ* to generate serpentine filopodia. (**e**) Mapping caspase-3 cleavage site PVD in p70S6K and PKC-*ζ* to generate constitutively active form devoid of auto-inhibitory domain (AID) but with intact kinase domain (KD/PKD). The epitopes for the corresponding antibodies are also shown to explain why these antibodies are capable of detecting p45S6K and PKM-*ζ*. (**f**) Western blotting showing that blebbishields retain phosphorylation of ribosomal S6 protein, a target of p70S6K (left). Isolated RT4P blebbishields form spheres (indicating transformation), and inhibition of S6K with BI-D1870 abolishes transformation from blebbishields (right). Cl., cleaved; Ph., phosphorylated; Tot., total.

In RT4P cells, we identified five novel constitutive interactions of RalGDS-RBD (Ral binding domain) with cdc42 (filopodia initiator^16^), PKC-*ζ* (filopodia regulator, a link between VEGF and PMA to regulate polarity^33,34^), internal but not surface VEGFR2 (surface VEGFR2 is heavily glycosylated and hence is >230 kDa^1^), E-cadherin (cell adhesion regulator^35^), and full-length and caspase-3 cleaved *β*-catenin (a transcription factor that can transcribe VEGF and also links E-cadherin with p120 catenin and cytoskeleton^35,36^). We also detected a constitutive interaction of RalGDS-RBD with K-Ras, a transformation and tumorigenesis driver^37,38^ previously known to interact with RalGDS.^39^ However, in blebbishields, RalGDS-RBD selectively lost interactions with E-cadherin and full-length *β*-catenin (Figure 6a).

K-Ras, *β*-catenin, and VEGFR2 are well-known regulators of transformation, VEGF transcription, and VEGFR2 transcription respectively.^36,40^ Hence, we propose a model (Figure 6b) in which RalGDS, along with its interaction partners K-Ras, PKC-*ζ*, VEGFR2, cdc42, and *β*-catenin, is constitutively anchored to E-cadherin at the plasma membrane in a locked state and in which apoptotic caspase-3-mediated cleavage of *β*-catenin (and possibly *α*/p120-catenin) releases RalGDS and its interaction partners to proceed with the downstream RalA-RalBP1 endocytic/tumorigenic cascade. Taken together, these data demonstrated why blebbishields exhibit robust endocytosis and how endocytosis may stimulate VEGF autocrine signaling to strengthen VEGF-mediated transformation from blebbishields.

### Cleavage of p70S6K and PKC-*ζ* generates p45S6K and PKM-*ζ* to facilitate serpentine filopodia formation initiated by cdc42 and PAK-1

Although we found that cdc42 interacts with RalGDS during the endocytosis cascade, it is not known what drives the formation of blebbishield-specific serpentine filopodia. To answer this question, we examined specific interactions of filopodia regulators with PAK-1-CRIB domain and cdc42, two key elements of the filopodia formation. We found that p45S6K (caspase-3-cleaved p70S6K) and PKM-*ζ* (cleaved PKC-*ζ*) interacted with the PAK-1-CRIB domain specifically in blebbishields (Figures 6c and d). In the case of p70S6K, the caspase-3 cleavage site was previously identified as an unorthodox TPVD site.^41^ We found a PVD site in PKC-*ζ* similar to the p70S6K TPVD site. Caspase-3 cleavage can result in the generation of constitutively active kinases of p45S6K and PKM-*ζ* because of the removal of their auto-inhibitory domains (Figure 6e). Interestingly, PKC-*ζ* and p70S6K are known to regulate filopodia.^42,43^ Hence, it is conceivable that p45S6K and PKM-*ζ* interaction with PAK-1-CRIB domain and cdc42 regulates formation of specialized serpentine filopodia. On the other hand, p70S6K is known to regulate translation and VEGF expression by phosphorylating ribosomal S6 proteins.^44^ Blebbishields retain phosphorylated ribosomal S6 proteins, and inhibiting S6Ks using BI-D1870 abrogated transformation from blebbishields (Figure 6f). Taken together, these data demonstrated that filopodia formation and protein translation are vital for blebbishield formation and transformation from blebbishields.

## Concluding remarks

**Figure 7:**
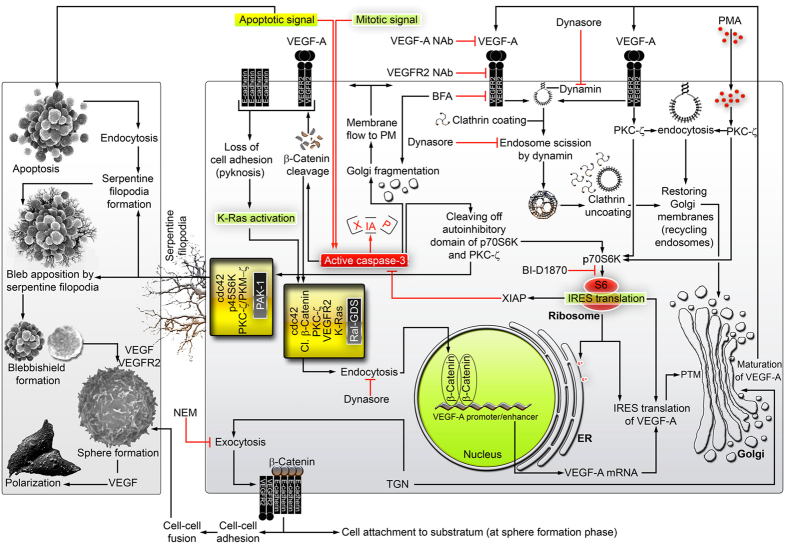
Schematic showing how endocytosis, filopodia, VEGF/VEGFR2 regulate membrane traffic to execute the blebbishield emergency program. Left panel depicts the cellular level aspects of the blebbishield emergency program whereas right panel depicts organelle, and molecular-level events of the blebbishield emergency program. Molecules listed within yellow boxes are demonstrated to interact with the molecules identified by black boxes within. Red lines indicate events leading to either apoptosis or blocking survival whereas black lines indicate survival paths. NEM, *N*-ethylmaleimide; TGN, *trans*-Golgi network; BFA, brefeldin-A; NAb, neutralizing antibody; Cl. *β*-catenin, cleaved *β*-catenin; PMA, phorbol-myristic acetate; ER, endoplasmic reticulum; PM, plasma membrane; PTM, post-translational modification.

Resumption of endocytosis is the key aspect well played by mitotic cells.^7^ Our findings here demonstrate that in apoptotic cells, endocytosis leads to the formation of blebbishields to initiate resurrection (Figure 7). Although rounding-off in mitotic cells and pyknosis in apoptotic cells are known to result in loss of E-cadherin-mediated cell–cell contacts, we found no detectable endocytosis in freshly isolated mitotic cells (Figures 3b and c), suggesting that this loss of cell–cell contacts might not trigger endocytosis. Hence, endocytosis must be initiated after loss of RalGDS–E-cadherin interaction. In support of this notion, only cells in late stages of mitosis, such as anaphase to telophase, exhibited fusion (Figure 3c). We conclude that in mitotic cells, transient arrest of endocytosis promotes accumulation of endomembranes (such as Golgi membranes) at the cell surface, whereas in apoptotic cells, blebbing brings endomembranes to the cell surface. Golgi membranes are well known for their fusion behavior, especially during reassembly of the Golgi apparatus after mitosis.^45^ Fusion behavior of blebbishields and mitotic cells is indeed regulated by Golgi membranes, as indicated by our findings such as, fusion of blebbishields with mitotic cells with PKH-labeled membranes accumulated at perinuclear region, fusion of mitotic cells to each other with their Golgi membranes exposed at the surface, co-localization of VEGFR2 with PKH-labeled membranes with trans-Golgi network marker syntaxin-6 and inhibition of blebbishield-mediated transformation with the use of drugs affecting Golgi integrity.

Initiation of endocytosis in blebbishields is explained by the loss of RalGDS interaction with full-length *β*-catenin and E-cadherin. E-cadherin, *α*-catenin, and *β*-catenin are well known to anchor the cytoskeleton with the plasma membrane. Hence, it is conceivable that RalGDS and the identified novel and known interaction partners are anchored to the plasma membrane by catenin–E-cadherin interaction. Loss of RalGDS interaction with E-cadherin and full-length *β*-catenin can release RalGDS from E-cadherin lock to initiate endocytosis because RalGDS downstream effectors RalA and RalBP1 are bona fide endocytosis regulators also required for tumorigenesis from Ras (Figure 7).^11^ Our identification of K-Ras–RalGDS-RBD interaction in blebbishields further supports this notion. Although we showed that VEGF signaling drives transformation from blebbishields, our identification of internal VEGFR2 interaction with RalGDS further demonstrates that VEGFR2 transduces signals through a RalGDS-RalA-RalBP1-K-Ras axis to regulate transformation from blebbishields (Figure 7).

Formation of filopodia to construct blebbishields from apoptotic bodies is explained by the fact that endocytosis drives filopodia formation^19^ and filopodia formation is essential for membrane fusion.^17^ Our identification of cdc42 interaction with RalGDS further strengthens this concept that filopodia formation and endocytosis are co-regulated by RalGDS. We attribute PAK1–CRIB interaction with p45S6K, PKM-*ζ*, and filopodia initiator cdc42 during blebbishield formation to the reason behind serpentine filopodia formation to provide a mechanical role in the construction of blebbishields from apoptotic bodies. Caspase-3 is known to cleave-off p70S6K auto-inhibitory domain^41^ to generate constitutively active p45S6K and hence might play a role in generation of constitutively active PKM-*ζ* from PKC-*ζ* as it has a similar PVD site (Figure 6e).

Together, our findings demonstrate that endocytosis is required for blebbishield formation after apoptosis through serpentine-filopodia-mediated apposition of apoptotic bodies to facilitate membrane fusion and that a RalGDS-endocytosis-filopodia-VEGF-VEGFR2-K-Ras-cdc42-p70S6K axis drives the blebbishield emergency program (Figure 7).

## Materials and methods

### Cells, cell maintenance, and blebbishield isolation

Human RT4 bladder cancer cells (American Type Culture Collection; HTB-2, referred to as RT4 parental (RT4P) in this study) and RT4v6 cells (described previously^1^) were cultured in MEM with 10% fetal bovine serum, L-glutamine, pyruvate, nonessential amino acids, vitamins, penicillin, and streptomycin. 293T cells were maintained in 10% DMEM and experimented in MEM for fusion assays. Blebbishields were isolated as described previously^1^ with blebbishield ejection medium containing 1 *μ*M cisplatin and 20 mM lithium chloride.

### Plasmids

Bacterial expression plasmid pGEX-RalGDS-RBD^46^ (C-terminal 97 residues) was a gift from Dr. Lawrence A. Quilliam, Indiana University School of Medicine, Indiana, USA. Bacterial expression plasmid pGEX-PAK1-CRIB^47^ was a gift from Dr. Philippe Chavrier, Institut Curie, Paris, France. The pGEX-4 T control plasmid was a gift from Dr. Santosh Chauhan, the University of New Mexico, USA.

Eukaryotic expression plasmid pcDNA3-Golgi-CFP^48^ (Addgene: 14873) was a gift from Dr. Alexandra C. Newton, University of California San Diego, California, USA.

### Reagents

Nocodazole (M1404), PKH67 (green) (PKH67GL-1KT), PKH26 (red) (PKH26GL-1KT), brefeldin-A (B7651, used at 5 *μ*g/ml), N-ethylmaleimide (E3876, used at 50 *μ*M), LiCl2 (L-4408), PMA (used at 100 nM; P8139), and dynasore (D7693, used at 100 *μ*M) were purchased from Sigma (St. Louis, MO, USA). TNF-*α* (210-TA) (used at 17 ng/ml), VEGF (used at 19 ng/ml; 293-VE), and neutralizing antibodies to VEGF (used at 1 *μ*g/ml; MAB293) and VEGFR2 (used at 1 *μ*g/ml; MAB3572) were purchased from R&D Systems (Minneapolis, MN, USA). Smac mimetic TL-32711 (A-1901) (used at 100 nM) was purchased from Active Biochem (Maplewood, NJ, USA). Cisplatin (NDC 0015-3220-22) was purchased from Bristol Laboratories (Princeton, NJ, USA). Antibodies to VEGF (for WB: Sc-7269), PKC-*ζ* (Sc-17781), K-Ras (Sc-30), and VEGFR2 (for WB: Sc-504) were purchased from Santa Cruz Biotechnology (Santa Cruz, CA, USA). Antibodies to E-cadherin (3195), p70S6K (2708), phospho-S6 ribosomal protein (Ser-235/236) (2211), S6 ribosomal protein (2317), syntaxin-6 (2869), and cdc42 (2466) were purchased from Cell Signaling Technology (Beverly, MA, USA). Antibody to *β*-catenin (610153) was purchased from BD Transduction Laboratories (Lexington, KY, USA). BI-D1870 (used at 5 *μ*M; S2843) was purchased from Selleckchem (Houston, TX, USA).

### Apoptotic body counting

For counting of individual apoptotic bodies, RT4v6 cells were treated with 100 nM TL-32711 and 17 ng/ml TNF-*α* with or without 100 *μ*M dynasore. At 7 h after treatment, the apoptotic bodies were counted.

### Transient transfection

HEK-293T cells were transiently transfected with Golgi-CFP plasmid using the calcium phosphate-BES method as described previously,^49^ and the cells were imaged for mitotic figures at 24 h after DNA addition. CFP fluorescence was pseudocolored to red.

### Preparation of mitotic cells

RT4 cells (1×10^7^) were arrested at mitotic phase using 400 ng/ml nocodazole treatment for 4 h (longer incubations may induce apoptosis) in 15 ml of 15% FBS containing MEM, and the mitotic cells were then collected by the ‘mitotic shake-off’ method.^50^

### Blebbishield–mitotic cell fusion assays and tracking of surface membranes

Blebbishields and mitotic cells were isolated in parallel and were washed with 10 ml of serum-free MEM at 1200 rpm for 3 min and resuspended in diluent-C of PKH membrane linker kits. Blebbishields were linked with PKH26 (red) and mitotic cells were linked with PKH67 (green) for 5 min. The remainder of the staining procedure was done according to the manufacturer’s instructions. The PKH-linked blebbishields and mitotic cells were mixed in equal proportions (1 : 1) in complete MEM and were plated in 6-well plates and imaged at 16–24 h.

### Scanning electron microscopy of blebbishields, 2D imaging, and time-lapse microscopy

Scanning electron microscopy of blebbishields and 2D and time-lapse imaging studies were performed as described previously.^1^ To evaluate endocytosis of PKH26-stained membranes, blebbishields were quick-fixed (4% paraformaldehyde+0.3% glutaraldehyde in PBS for 15 min at room temperature), washed with PBS thrice and with diluent-C once, stained with PKH26 (red), and imaged.

### Time-lapse imaging

To evaluate the role of endocytosis in blebbishield formation, RT4P cells were treated with 100 *μ*M dynasore in blebbishield ejection medium, and the apoptotic cells were subjected to time-lapse imaging for observation of fusion events. To evaluate vesicle trafficking, blebbishields were labeled with PKH26 and allowed to form spheres for 16 h. The spheres were then subjected to time-lapse imaging. To evaluate the effect of VEGF on cell attachment after trypsinization, RT4P cells were trypsinized and incubated with or without rh-VEGF (19 ng/ml) containing complete MEM and subjected to time-lapse imaging. To evaluate the effect of PMA on polarization of spheres, spheres were generated from blebbishields for 16 h and subjected to time-lapse imaging after addition of 100 ng/ml PMA.

### Lipo-immunofluorescence of spheres

Blebbishields were isolated and washed with serum-free MEM, and the surface membrane lipids were linked with PKH26 for 5 min in diluent-C. Excess PKH26 was then washed off, and blebbishields were allowed to form spheres in 12-well plates for 16 h. Unattached blebbishields were washed off using serum-free MEM, and attached spheres were fixed using 4% PFA in PBS at room temperature for 20 min. The spheres were then processed for immunofluorescence to identify co-localization of VEGFR2 or syntaxin-6 with PKH26-labeled membranes.

### Immunofluorescence of blebbishields and RT4P cells

Blebbishields were isolated as described above, or cells in 24-well plates were washed with PBS and fixed using 4% PFA in PBS for 20 min at room temperature, washed with PBS thrice (pelleted at 500 rpm for 2 min for blebbishields), blocked with 1% BSA in PBS with 0.3% tritin-X100 for 30 min, and incubated in primary antibodies (in blocking buffer) for 1 hr at room temperature. Blebbishields or cells were washed with PBS thrice and incubated with secondary antibodies conjugated with Alexa-555 or Alexa-488 for 1 h at room temperature. Blebbishields or cells were washed with PBS and then imaged.

### Blebbishield-mediated transformation (sphere formation) assays

Blebbishields were isolated from RT4P cells and allowed to form spheres in 6-well plates in the presence or absence of various agents in complete MEM as indicated in the figures for 16–24 h. The floating cells were washed off, and the attached spheres were counted by scanning the well from end to end.

For VEGF and VEGFR2 neutralizing antibody tests, 1 *μ*g/ml of each antibody was used per 100 000 blebbishields/ml during sphere formation phase (higher blebbishield density requires more antibodies).

### GST-PAK-1-CRIB and GST-RalGDS-RBD pulldown assays

DH5*α* strains were transformed with pGEX constructs; induced with 1 mM IPTG (I6758; Sigma) for 4 h at 37 °C; lysed using sonication (output: 8; 15-s bursts, twice) in bacteria lysis buffer (20 mM HEPES (pH 7.5), 120 mM NaCl, 10% (v/v) glycerol, 2 mM EDTA, 1 mM DTT, 0.5% NP-40, 1×-protease inhibitor cocktail, and 1 mM PMSF); clarified at 13 000 r.p.m. for 10 min; bound to glutathione sepharose-CL4B for 1 h at 4 °C; washed six times using bacteria lysis buffer; and then incubated with 200 *μ*g/200 *μ*l lysates prepared in whole cell lysis buffer (50 mM Tris-HCl (pH 7.4), 150 mM NaCl, 5 mM EDTA, 25 mM NaF, 1% Triton-X 100, 1% NP-40, 0.1 mM Na_3_VO_4_, 12.5 mM *β*-glycerophosphate, 1 mM PMSF, and 1×-complete protease inhibitor cocktail)+200 *μ*l of magnesium-containing lysis buffer (25 mM HEPES (pH 7.5), 150 mM NaCl, 1% (v/v) NP-40, 0.25% (w/v) sodium deoxycholate, 10% glycerol, 20 mM MgCl2, 1 mM EDTA, 1×-protease inhibitor cocktail, and 1 mM PMSF) for 1 h at 4 °C. Pulldown complexes were washed four times with magnesium-containing lysis buffer before being subjected to SDS-PAGE and immunoblotting. The protein interactions from this publication have been submitted to IMEx consortium (http://www.imexconsortium.org) through IntAct^51^ and assigned the identifier IM-24378 (EBI-11165786, EBI-11165886, EBI-11165907, EBI-11165919, EBI-11165949, EBI-11165963, EBI-11165989, EBI-11166003, EBI-11166025, EBI-11166037).

### Western immunoblotting

Cells/blebbishields were lysed using whole cell lysis buffer (described in the preceding section on GST-pulldown assays) for 40 min with vortexing every 10 min. The lysates were clarified at 13 000 r.p.m. for 10 min, quantified using BCA assay, and subjected to SDS-PAGE and transfer to nitrocellulose membrane before probing with antibodies. The signals were developed using enhanced chemiluminescence.

### Statistical analyses

Statistical analyses were performed using Microsoft Excel 2010. Statistical significance was determined on the basis of Student’s *t*-test with two-tailed distribution and two-sample unequal variance, and *P* values below 0.05 were considered significant. Error bars represent S.E.M.

## References

[bib1] Jinesh GG , Choi W , Shah JB , Lee EK , Willis DL , Kamat AM . Blebbishields, the emergency program for cancer stem cells: sphere formation and tumorigenesis after apoptosis. Cell Death Differ 2013; 20: 382–395. 23175184 10.1038/cdd.2012.140 PMC3569985

[bib2] Maugeri-Sacca M , Vici P , Di Lauro L , Barba M , Amoreo CA , Gallo E et al. Cancer stem cells: are they responsible for treatment failure? Future Oncol 2014; 10: 2033–2044. 25396775 10.2217/fon.14.126

[bib3] Goodwin Jinesh G , Willis DL , Kamat AM . Bladder cancer stem cells: biological and therapeutic perspectives. Curr Stem Cell Res Ther 2014; 9: 89–101. 24236543 10.2174/1574888x08666131113123051

[bib4] Chiu R , Novikov L , Mukherjee S , Shields D . A caspase cleavage fragment of p115 induces fragmentation of the Golgi apparatus and apoptosis. J Cell Biol 2002; 159: 637–648. 12438416 10.1083/jcb.200208013 PMC2173109

[bib5] Lowe M , Rabouille C , Nakamura N , Watson R , Jackman M , Jamsa E et al. Cdc2 kinase directly phosphorylates the cis-Golgi matrix protein GM130 and is required for Golgi fragmentation in mitosis. Cell 1998; 94: 783–793. 9753325 10.1016/s0092-8674(00)81737-7

[bib6] Fielding AB , Willox AK , Okeke E , Royle SJ . Clathrin-mediated endocytosis is inhibited during mitosis. Proc Natl Acad Sci U S A 2012; 109: 6572–6577. 22493256 10.1073/pnas.1117401109 PMC3340072

[bib7] Schweitzer JK , Burke EE , Goodson HV , D'Souza-Schorey C . Endocytosis resumes during late mitosis and is required for cytokinesis. J Biol Chem 2005; 280: 41628–41635. 16207714 10.1074/jbc.M504497200

[bib8] Rosse C , L'Hoste S , Offner N , Picard A , Camonis J . RLIP, an effector of the Ral GTPases, is a platform for Cdk1 to phosphorylate epsin during the switch off of endocytosis in mitosis. J Biol Chem 2003; 278: 30597–30604. 12775724 10.1074/jbc.M302191200

[bib9] Raucher D , Sheetz MP . Membrane expansion increases endocytosis rate during mitosis. J Cell Biol 1999; 144: 497–506. 9971744 10.1083/jcb.144.3.497 PMC2132908

[bib10] Austin CD , Lawrence DA , Peden AA , Varfolomeev EE , Totpal K , De Maziere AM et al. Death-receptor activation halts clathrin-dependent endocytosis. Proc Natl Acad Sci U S A 2006; 103: 10283–10288. 16801533 10.1073/pnas.0604044103 PMC1482799

[bib11] Lim KH , Baines AT , Fiordalisi JJ , Shipitsin M , Feig LA , Cox AD et al. Activation of RalA is critical for Ras-induced tumorigenesis of human cells. Cancer Cell 2005; 7: 533–545. 15950903 10.1016/j.ccr.2005.04.030

[bib12] Han K , Kim MH , Seeburg D , Seo J , Verpelli C , Han S et al. Regulated RalBP1 binding to RalA and PSD-95 controls AMPA receptor endocytosis and LTD. PLoS Biol 2009; 7: e1000187. 19823667 10.1371/journal.pbio.1000187 PMC2730530

[bib13] Matsubara K , Kishida S , Matsuura Y , Kitayama H , Noda M , Kikuchi A . Plasma membrane recruitment of RalGDS is critical for Ras-dependent Ral activation. Oncogene 1999; 18: 1303–1312. 10022812 10.1038/sj.onc.1202425

[bib14] Mosesson Y , Mills GB , Yarden Y . Derailed endocytosis: an emerging feature of cancer. Nat Rev Cancer 2008; 8: 835–850. 18948996 10.1038/nrc2521

[bib15] Welch MD , Mullins RD . Cellular control of actin nucleation. Annu Rev Cell Dev Biol 2002; 18: 247–288. 12142287 10.1146/annurev.cellbio.18.040202.112133

[bib16] Mattila PK , Lappalainen P . Filopodia: molecular architecture and cellular functions. Nat Rev Mol Cell Biol 2008; 9: 446–454. 18464790 10.1038/nrm2406

[bib17] Millard TH , Martin P . Dynamic analysis of filopodial interactions during the zippering phase of Drosophila dorsal closure. Development 2008; 135: 621–626. 18184725 10.1242/dev.014001 PMC2440488

[bib18] Vasioukhin V , Bauer C , Yin M , Fuchs E . Directed actin polymerization is the driving force for epithelial cell-cell adhesion. Cell 2000; 100: 209–219. 10660044 10.1016/s0092-8674(00)81559-7

[bib19] Bu W , Chou AM , Lim KB , Sudhaharan T , Ahmed S . The Toca-1-N-WASP complex links filopodial formation to endocytosis. J Biol Chem 2009; 284: 11622–11636. 19213734 10.1074/jbc.M805940200 PMC2670167

[bib20] Lanzetti L , Di Fiore PP . Endocytosis and cancer: an 'insider' network with dangerous liaisons. Traffic 2008; 9: 2011–2021. 18785924 10.1111/j.1600-0854.2008.00816.x

[bib21] Baker J , Garrod D . Epithelial cells retain junctions during mitosis. J Cell Sci 1993; 104: 415–425. 7685036 10.1242/jcs.104.2.415

[bib22] Macia E , Ehrlich M , Massol R , Boucrot E , Brunner C , Kirchhausen T . Dynasore, a cell-permeable inhibitor of dynamin. Dev Cell 2006; 10: 839–850. 16740485 10.1016/j.devcel.2006.04.002

[bib23] Kirchhausen T , Macia E , Pelish HE . Use of dynasore, the small molecule inhibitor of dynamin, in the regulation of endocytosis. Methods Enzymol 2008; 438: 77–93. 18413242 10.1016/S0076-6879(07)38006-3 PMC2796620

[bib24] Jinesh GG , Chunduru S , Kamat AM . Smac mimetic enables the anticancer action of BCG-stimulated neutrophils through TNF-alpha but not through TRAIL and FasL. J Leukoc Biol 2012; 92: 233–244. 22517918 10.1189/jlb.1211623 PMC3382315

[bib25] Jinesh GG , Kamat AM . Redirecting neutrophils against bladder cancer cells by BCG and Smac mimetic combination. Oncoimmunology 2012; 1: 1161–1162. 23170264 10.4161/onci.20928 PMC3494630

[bib26] Robinson LJ , Aniento F , Gruenberg J . NSF is required for transport from early to late endosomes. J Cell Sci 1997; 110: 2079–2087. 9378758 10.1242/jcs.110.17.2079

[bib27] Wang E , Pennington JG , Goldenring JR , Hunziker W , Dunn KW . Brefeldin A rapidly disrupts plasma membrane polarity by blocking polar sorting in common endosomes of MDCK cells. J Cell Sci 2001; 114: 3309–3321. 11591819 10.1242/jcs.114.18.3309

[bib28] Manickam V , Tiwari A , Jung JJ , Bhattacharya R , Goel A , Mukhopadhyay D et al. Regulation of vascular endothelial growth factor receptor 2 trafficking and angiogenesis by Golgi localized t-SNARE syntaxin 6. Blood 2011; 117: 1425–1435. 21063020 10.1182/blood-2010-06-291690 PMC3056478

[bib29] Gonzalez-Garcia A , Pritchard CA , Paterson HF , Mavria G , Stamp G , Marshall CJ . RalGDS is required for tumor formation in a model of skin carcinogenesis. Cancer Cell 2005; 7: 219–226. 15766660 10.1016/j.ccr.2005.01.029

[bib30] Ohta Y , Suzuki N , Nakamura S , Hartwig JH , Stossel TP . The small GTPase RalA targets filamin to induce filopodia. Proc Natl Acad Sci USA 1999; 96: 2122–2128. 10051605 10.1073/pnas.96.5.2122 PMC26747

[bib31] Sugihara K , Asano S , Tanaka K , Iwamatsu A , Okawa K , Ohta Y . The exocyst complex binds the small GTPase RalA to mediate filopodia formation. Nat Cell Biol 2002; 4: 73–78. 11744922 10.1038/ncb720

[bib32] Cullen PJ , Lockyer PJ . Integration of calcium and Ras signalling. Nat Rev Mol Cell Biol 2002; 3: 339–348. 11988768 10.1038/nrm808

[bib33] Wang HR , Zhang Y , Ozdamar B , Ogunjimi AA , Alexandrova E , Thomsen GH et al. Regulation of cell polarity and protrusion formation by targeting RhoA for degradation. Science 2003; 302: 1775–1779. 14657501 10.1126/science.1090772

[bib34] Shih SC , Mullen A , Abrams K , Mukhopadhyay D , Claffey KP . Role of protein kinase C isoforms in phorbol ester-induced vascular endothelial growth factor expression in human glioblastoma cells. J Biol Chem 1999; 274: 15407–15414. 10336429 10.1074/jbc.274.22.15407

[bib35] Ishiyama N , Lee SH , Liu S , Li GY , Smith MJ , Reichardt LF et al. Dynamic and static interactions between p120 catenin and E-cadherin regulate the stability of cell-cell adhesion. Cell 2010; 141: 117–128. 20371349 10.1016/j.cell.2010.01.017

[bib36] Li J , Mizukami Y , Zhang X , Jo WS , Chung DC . Oncogenic K-ras stimulates Wnt signaling in colon cancer through inhibition of GSK-3beta. Gastroenterology 2005; 128: 1907–1918. 15940626 10.1053/j.gastro.2005.02.067

[bib37] Malumbres M , Barbacid M . RAS oncogenes: the first 30 years. Nat Rev Cancer 2003; 3: 459–465. 12778136 10.1038/nrc1097

[bib38] Park MT , Kim MJ , Suh Y , Kim RK , Kim H , Lim EJ et al. Novel signaling axis for ROS generation during K-Ras-induced cellular transformation. Cell Death Differ 2014; 21: 1185–1197. 24632950 10.1038/cdd.2014.34 PMC4085525

[bib39] Wang J , Huo K , Ma L , Tang L , Li D , Huang X et al. Toward an understanding of the protein interaction network of the human liver. Mol Syst Biol 2011; 7: 536. 21988832 10.1038/msb.2011.67 PMC3261708

[bib40] Domingues I , Rino J , Demmers JA , de Lanerolle P , Santos SC . VEGFR2 translocates to the nucleus to regulate its own transcription. PLoS One 2011; 6: e25668. 21980525 10.1371/journal.pone.0025668 PMC3182252

[bib41] Dhar R , Persaud SD , Mireles JR , Basu A . Proteolytic cleavage of p70 ribosomal S6 kinase by caspase-3 during DNA damage-induced apoptosis. Biochemistry 2009; 48: 1474–1480. 19191576 10.1021/bi801840s PMC2701466

[bib42] Ip CK , Cheung AN , Ngan HY , Wong AS . p70 S6 kinase in the control of actin cytoskeleton dynamics and directed migration of ovarian cancer cells. Oncogene 2011; 30: 2420–2432. 21258406 10.1038/onc.2010.615

[bib43] Loitto VM , Huang C , Sigal YJ , Jacobson K . Filopodia are induced by aquaporin-9 expression. Exp Cell Res 2007; 313: 1295–1306. 17346701 10.1016/j.yexcr.2007.01.023

[bib44] Bian CX , Shi Z , Meng Q , Jiang Y , Liu LZ , Jiang BH . P70S6K 1 regulation of angiogenesis through VEGF and HIF-1alpha expression. Biochem Biophys Res Commun 2010; 398: 395–399. 20599538 10.1016/j.bbrc.2010.06.080 PMC2928061

[bib45] Acharya U , Jacobs R , Peters JM , Watson N , Farquhar MG , Malhotra V . The formation of Golgi stacks from vesiculated Golgi membranes requires two distinct fusion events. Cell 1995; 82: 895–904. 7553850 10.1016/0092-8674(95)90269-4

[bib46] Asuri S , Yan J , Paranavitana NC , Quilliam LA . E-cadherin dis-engagement activates the Rap1 GTPase. J Cell Biochem 2008; 105: 1027–1037. 18767072 10.1002/jcb.21902 PMC2657844

[bib47] Niedergang F , Colucci-Guyon E , Dubois T , Raposo G , Chavrier P . ADP ribosylation factor 6 is activated and controls membrane delivery during phagocytosis in macrophages. J Cell Biol 2003; 161: 1143–1150. 12810696 10.1083/jcb.200210069 PMC2172982

[bib48] Gallegos LL , Kunkel MT , Newton AC . Targeting protein kinase C activity reporter to discrete intracellular regions reveals spatiotemporal differences in agonist-dependent signaling. J Biol Chem 2006; 281: 30947–30956. 16901905 10.1074/jbc.M603741200

[bib49] Kingston RE , Chen CA , Okayama H . Calcium phosphate transfection. Curr Protoc Immunol 2001; Chapter-10: Unit-10.13. 10.1002/0471142735.im1013s31 18432676

[bib50] Jackman J , O’Connor PM . Methods for synchronizing cells at specific stages of the cell cycle. Curr Protoc Cell Biol 2001; Chapter-8: Unit-8.3. 10.1002/0471143030.cb0803s00 18228388

[bib51] Orchard S , Ammari M , Aranda B , Breuza L , Briganti L , Broackes-Carter F et al. The MIntAct project--IntAct as a common curation platform for 11 molecular interaction databases. Nucleic Acids Res 2014; 42: D358–D363. 24234451 10.1093/nar/gkt1115 PMC3965093

