# Supplementary material for: Endocytosis and serpentine filopodia drive blebbishield-mediated resurrection of apoptotic cancer stem cells
Source: Cell Death Discov. 2016 Jan 25;2:15069–. doi: 10.1038/cddiscovery.2015.69 (PMC4876976; doi:10.1038/cddiscovery.2015.69)
Supplement: Supplementary Information [file cddiscovery201569-s1.doc]

**Supplemental movie legends**

**Supplemental movie S1. Blebbishield surface membranes are transported as vesicles inside spheres**

Blebbishields were isolated from RT4P cells using blebbishield ejection medium, stained for surface membranes using PKH26 dye, and allowed for sphere formation (transformation) and imaged. Note the vesicular movements within spheres.

**Supplemental movie S2. Dynasore prevents blebbishield formation by promoting individual apoptotic body formation**

RT4P cells were induced to undergo apoptosis using TNF-α plus Smac mimetic TL-32711 in the presence of dynasore, and were imaged by time-lapse microscopy to observe fusion and blebbishield formation. Note that the vesicles fail to fuse or to form blebbishields.

**Supplemental movie S3. VEGF is not required for attachment of RT4P cells to substratum after trypsinization**

RT4P cells were trypsinized and incubated with or without VEGF neutralizing antibodies and imaged simultaneously by time-lapse imaging. Note that VEGF neutralizing antibody does not inhibit or slowdown attachment of RT4P cells.

**Supplemental movie S4. PMA accelerates polarization of blebbishield derived spheres**

Blebbishields were isolated and allowed to form spheres for 16 hours. 100 ng/ml PMA was added and the spheres were subjected to time lapse microscopy to observe the speed of polarization.
